# Funny Lumps, Flaming Pheo, and a Broken Heart: A Rare Case of Pheochromocytoma

**DOI:** 10.7759/cureus.3646

**Published:** 2018-11-28

**Authors:** Khurram Butt, Saeed Ali, Zeeshan Sattar, Asad Ur Rahman, Jeremy R Burt

**Affiliations:** 1 Internal Medicine, Florida Hospital-Orlando, Orlando, USA; 2 Internal Medicine, Khyber Teaching Hospital, Peshawar, PAK; 3 Gastroenterology, Cleveland Clinic Florida, Weston, USA; 4 Radiology, Florida Hospital-Orlando, Orlando, USA

**Keywords:** pheochromocytoma, takotsubo, neurofibromatosis

## Abstract

Pheochromocytoma is the underlying etiology in 0.1% of hypertensive cases. However, it may be present in up to 5.7% of patients with neurofibromatosis I (NF1). The burst of catecholamines inherent in pheochromocytoma has significant effects on the mechanical and electrical activity of the myocardium. Different theories have been postulated for myocardial stunning in patients with pheochromocytoma that include microvascular spasm, impaired fatty acid metabolism, increased production of oxygen-derived free radicals and dynamic left ventricular mid-cavity obstruction. QT interval prolongation is seen in 16% to 35% of patients with pheochromocytoma. Takotsubo cardiomyopathy (TS) is now being increasingly identified and it may be responsible for up to 40% of cases of acute catecholamine cardiomyopathy. These manifestations may sometimes precede or cloud the typical triad of a headache, sweating, and tachycardia.

We herein present a case of a 42-year-old female with a unique combination of QT prolongation, torsades de pointes, and TS caused by pheochromocytoma in the background of NF1. All these complications are potentially reversible with the removal of the underlying adrenal tumor, underscoring the importance of a high suspicion for pheochromocytoma in patients with NF1.

## Introduction

Pheochromocytoma is a catecholamine-secreting tumor (CST) of the adrenal glands, which is a rare cause of secondary hypertension. It has a well-known association with neurofibromatosis type 1 (NF1) [1]. CST usually presents as a classic episodic triad of a headache, sweating, and palpitations. Prolonged QT interval and Takotsubo cardiomyopathy can be found in association with pheochromocytoma. We herein present a case that showed a constellation of NF1, Takotsubo cardiomyopathy (TS), and QT prolongation leading to polymorphic ventricular tachycardia that preceded the diagnosis of pheochromocytoma.

## Case presentation

A 42-year-old Caucasian female with a known history of NF1 presented to the emergency department with an episode of palpitations, flushing, pounding headache, numbness and tingling in both arms, and shortness of breath for the past two hours. The patient reported a similar episode two weeks prior, and a complete basic cardiac workup performed with an outpatient cardiologist was unremarkable. A loop recorder was implanted to detect the possible arrhythmias. The patient complained of these episodes lasting one to two hours every two to three months for the past two years. She denied any anxiety, stress, or any situational factors.

**Figure 1:**
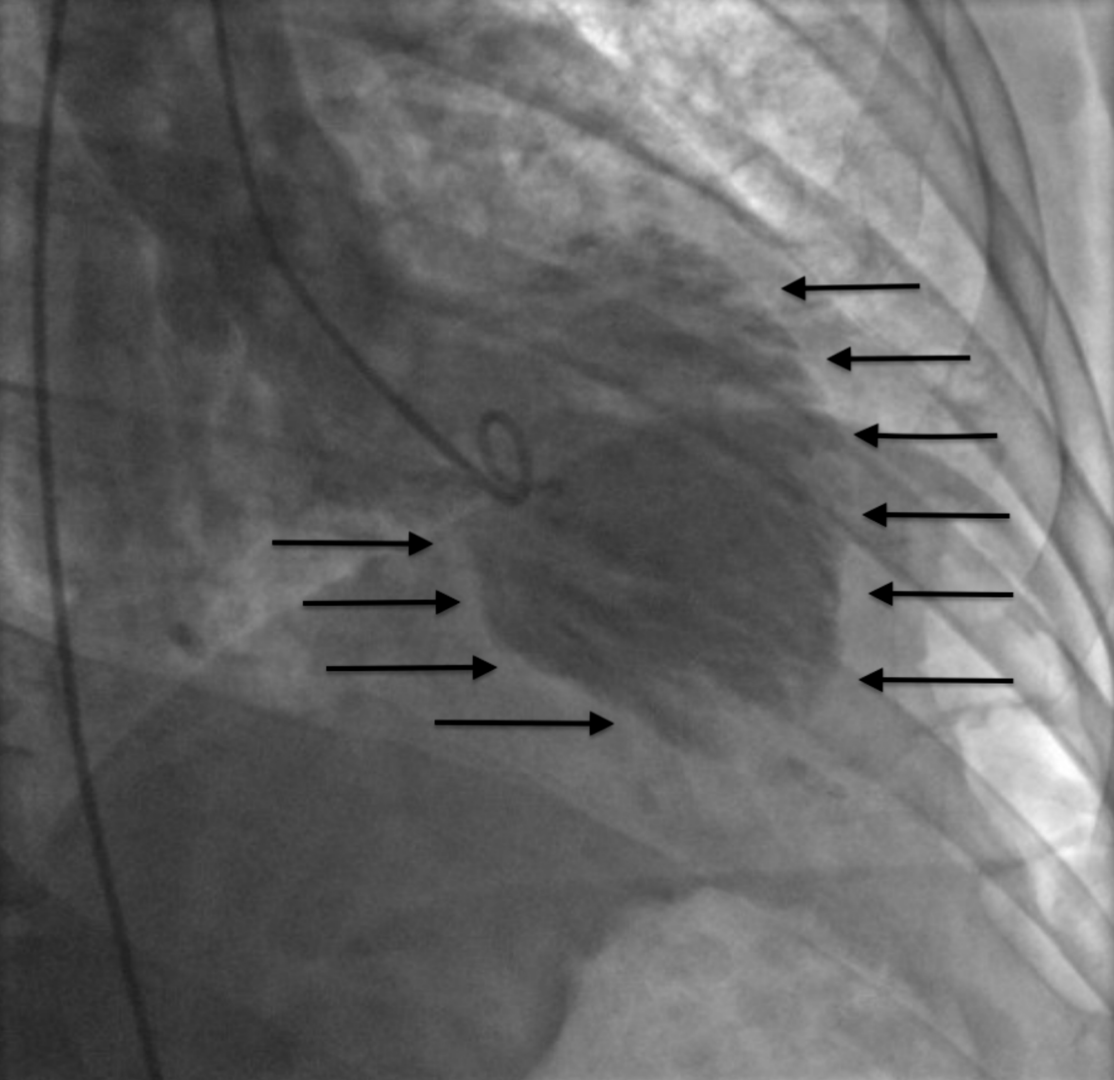
Left ventriculogram demonstrating classical mid to apical anterolateral and mid to apical inferior wall akinesis during ventricular systole (black arrows)

Her past medical history was significant for an episode of apparent ST-elevation myocardial infarction (STEMI) three years prior. During this hospitalization, her blood pressure was well controlled and her QTc interval was prolonged at 483 ms. Cardiac catheterization was performed emergently showing normal coronary anatomy without significant obstruction. However, the left ventriculogram revealed systolic apical ballooning with reasonable contractility at the cardiac base (Figure 1).

Her left ventricular ejection fraction (EF) was calculated at 25%, and she was diagnosed with TS. The patient was started on lisinopril and carvedilol per guideline-directed medical therapy (GDMT). Subsequently, she had complete recovery of cardiac function within three months as confirmed with the periodic follow-up echocardiography revealing EF improvement to 55%. Her blood pressure continued to be well controlled during this period. 

However, five months after the episode of TS, she had an asymptomatic episode of nonsustained polymorphic ventricular tachycardia (torsades de pointes) recorded on a loop recorder. She had prolongation of QTc interval in the baseline electrocardiogram (EKG). Her electrolytes were stable during this episode, and echocardiogram revealed an EF of 50% to 55%. As her EF had normalized, an implantable cardioverter-defibrillator (ICD) was not placed. She underwent electrophysiological studies twice that failed to reveal any etiology of arrhythmia.

**Figure 2:**
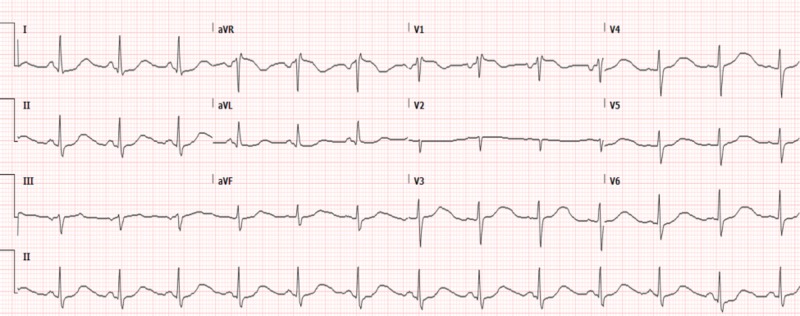
EKG prior to tumor resection demonstrating prolonged QT/QTc interval of 496/566 ms, respectively EKG: electrocardiogram

At the current presentation, physical examination was remarkable for a pulse rate of 101 beats per minute and blood pressure of 190/110 mmHg. She also had numerous cutaneous neurofibromas. Routine laboratory data showed a slightly elevated white blood cell count of 12.5 x 103/ul with normal hemoglobin and platelet counts. Her complete metabolic panel, electrolytes including magnesium, phosphorous, and thyroid-stimulating hormone levels were all within the normal limits. EKG showed sinus tachycardia with a QTc interval of 566 ms (Figure 2).

**Figure 3:**
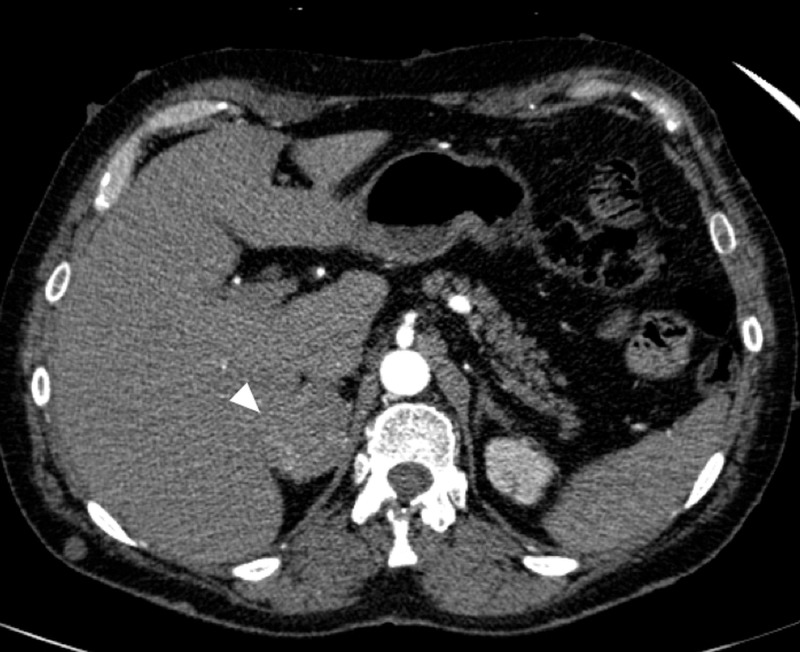
Axial contrast-enhanced CT of the upper abdomen showing the pheochromocytoma in the right adrenal gland (white arrowhead) CT: computed tomography

**Figure 4:**
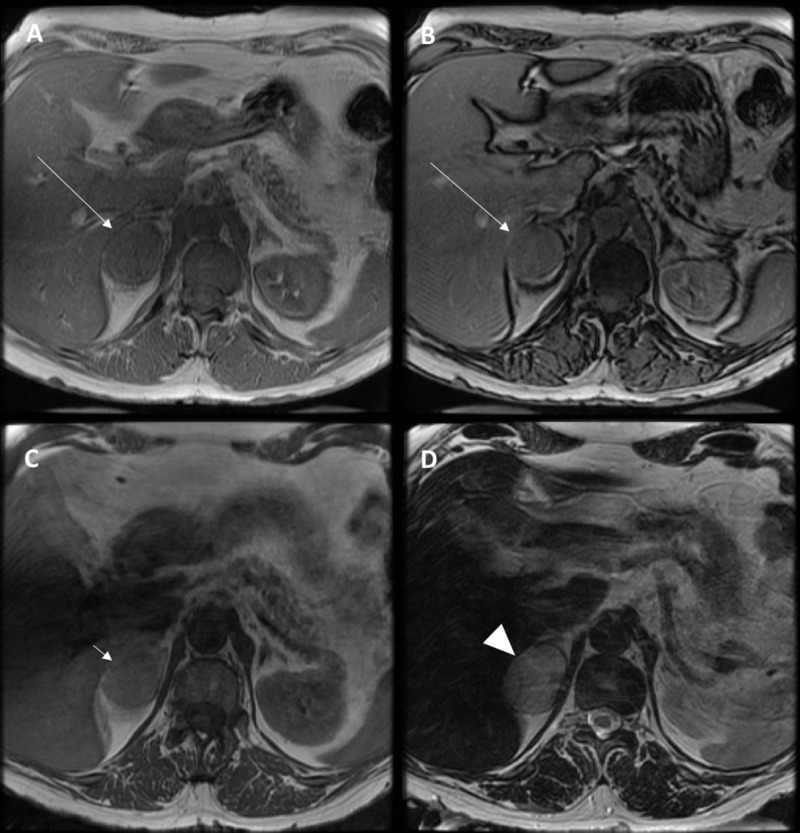
Axial MRI of the upper abdomen including (A) in-phase and (B) out-of-phase (FSPGR), (C) GRE T1, and FSE T2 (D) Note that the right adrenal mass does not decrease in signal on out-of-phase imaging (long white arrow), has an intermediate T1 signal (short white arrow) and a high T2 signal (white arrowhead). MRI: magnetic resonance imaging, FSPGR: fast spoiled gradient echo, GRE: gradient echo, FSE: fast spin echo

Loop recorder interrogation did not reveal any arrhythmia. A recent echo one month prior showed left ventricular ejection fraction of 60% to 65% with no significant structural abnormalities. With clinical suspicion of CST, further testing revealed elevated serum metanephrine at 7.90 nmol/L (normal: 0 to 0.49 nmol/L) and normetanephrine at 5.14 nmol/L (normal: 0-0.89 nmol/L). Diagnosis of CST was confirmed with 24-hour urine metanephrine levels of 5346 ug/day (normal <350 ug) and normetanephrine levels of 1817 ug/day (normal <650 ug). Subsequently, a computed tomography (CT) scan of the abdomen (Figure 3) was done that revealed a mass in the right adrenal gland, and the findings were confirmed with a magnetic resonance imaging of the abdomen, which revealed a 4.4 x 3.3-cm well-circumscribed heterogeneous fat-free mass in the right adrenal gland suspicious for a pheochromocytoma (Figure 4).

**Figure 5:**
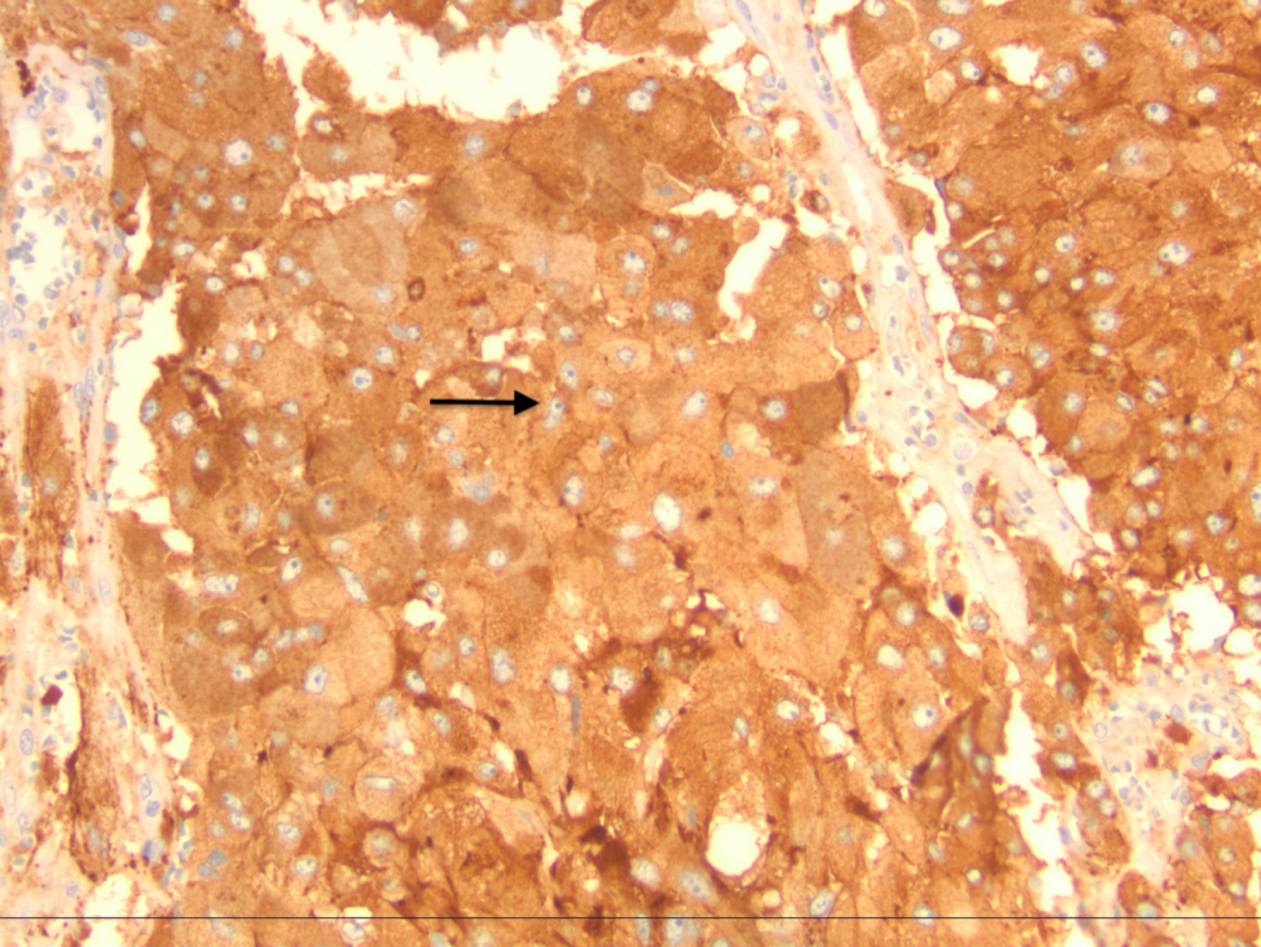
Synaptophysin immunohistochemistry x200 showing diffuse granular cytoplasmic staining consistent with pheochromocytoma

Testing for adrenal cortical hormones was normal. The patient was started on phenoxybenzamine for blood pressure control, and her home medication of labetalol was switched to sustained-release metoprolol. Her blood pressure was well controlled preoperatively. She underwent laparoscopic adrenalectomy, and her blood pressure remained controlled intra- and postoperatively. No additional medications were required for blood pressure control postoperatively. Histopathology revealed pheochromocytoma with positive synaptophysin staining extending 8 cm in the maximum dimension (Figure 5). There was no local invasion of the surrounding structures by tumor, and no distant metastases were found.

**Figure 6:**
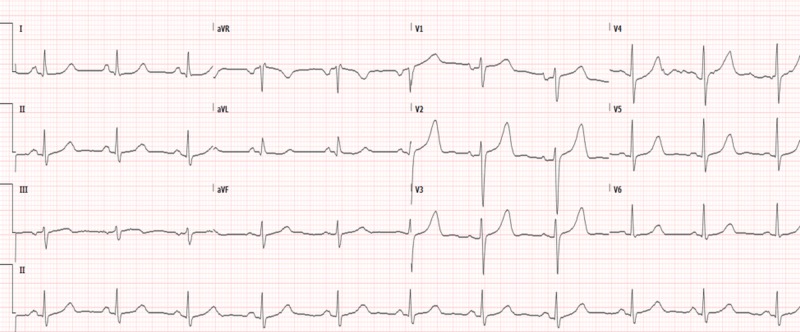
Post-operative EKG showing that QT/QTc interval is normalizing at 460/479 ms, respectively EKG: electrocardiogram

The patient recovered well from the surgery and on six months follow-up, her symptoms resolved completely with normalization of the QT interval (Figure 6).

## Discussion

CSTs are rare neoplasms with an annual incidence of 8 per million [2]. CST is a rare cause of secondary hypertension attributing to 0.2 percent of patients with hypertension [3]. Pheochromocytoma is most common in the fourth and fifth decades of life and is equally common in males and females.

Approximately a quarter to third cases of CST is part of a familial syndrome and may present as bilateral tumors. Hereditary CST typically presents at a younger age compared to the sporadic cases. Also, they are diagnosed earlier in the disease course due to routine surveillance and a high index of suspicion. The common disorders associated with CST are multiple endocrine neoplasias (MEN2), von Hippel Lindau (VHL) syndrome, and NF1. Approximate the frequency of pheochromocytoma in these syndromes is 50%, 10% to 20%, and 0.1% to 5.7%, respectively [1].

CSTs most commonly present with paroxysmal symptoms. The typical symptoms include an episodic headache, sweating, and palpitation. Other less common presentations include orthostatic hypotension, insulin resistance, secondary polycythemia, and cardiomyopathy. Rare cardiac presentations can be cardiac arrest, myocarditis, cardiogenic shock, and arrhythmias. With the recent advances and more use of advanced imaging techniques such as CT and MRI, more asymptomatic patients are diagnosed with pheochromocytoma [4].

Pheochromocytoma is increasingly recognized as a cause of TS like cardiomyopathy. A recent review of 80 published cases of pheochromocytoma-induced TS-like cardiomyopathy (Pheo-TS) compared them with 1750 published cases of TS. The review showed that patients with Pheo-TS are significantly younger than those with TS by an average of 20 years. Females are predominantly involved as in TS but significantly in a lower percentage (70% in Pheo-TS vs approximately 90% in TS). Also, almost a third of Pheo-TS cases presented with basal pattern TS (also called as inverse TS) compared to 2.2% in all cases of TS [5]. Basal pattern TS presents with a normal wall motion at apex but significantly reduced contractility of the left ventricular base and ballooning, which does not correlate to a certain vascular territory. Patients typically present with an acute coronary syndrome-like presentation with chest pain, dyspnea, palpitations, or hemodynamic instability. EKG may vary from being normal or sinus tachycardia to non-specific ST-T wave changes and ST elevation. Cardiac enzymes are usually elevated, and cardiac catheterizations reveal non-obstructive coronaries. Left ventriculography and transthoracic echocardiogram usually show the characteristic finding of systolic apical ballooning with a normally contracting base or vice versa. If the EF is <40%, GDMT is recommended and mostly results in the complete recovery of cardiac function as in our case.

CST and TS cardiomyopathy share pathophysiology with adrenergic cardiac toxicity due to the excessive release of catecholamines [6]. An acute catecholamine surge in pheochromocytoma causes flooding of the myocardial beta-receptors, resulting in transient myocardial stunning [7] and possibly some apoptosis. While in CST, patients are exposed to high levels of catecholamines chronically allowing time for myocardium to downregulate beta-receptors, desensitize, and adapt. Paroxysmal elevations of catecholamines may still cause similar effects on the myocardium as in TS, which may be the reason that very few patients with CST present with TS-like cardiomyopathy. CST can also lead to repeated TS-like cardiomyopathy as reported in the case reports [8].

CST has been found in associated with various arrhythmias, including sinus tachycardia, atrial fibrillation, supraventricular tachycardia, ventricular tachycardia, and sinus node dysfunction. It can cause QT prolongation, and a few cases have been reported in the literature where it led to torsades de pointes [9]. Pathophysiology may involve a similar mechanism as in cardiomyopathy with the overstimulation of the beta-receptors causing a positive chronotropic and inotropic effect. Also, chronically elevated catecholamines have less effect on myocardium than acute elevations, which may be the reason for less frequent ventricular arrhythmias in CST.

## Conclusions

We present herein a case of NF1 with pheochromocytoma, diagnosis of which was delayed by at least a couple of years. High-risk patients including those with familial syndromes or a family history of CST who are presenting with QT prolongation, unexplained cardiac arrhythmia, or TS-like syndrome should be screened for CST. The tumor should be resected whenever feasible. Pheochromocytoma carries about 10% risk of metastasis that can have a delayed presentation as well, so patients need a regular follow-up.
